# Biomechanical Pole Vault Patterns Were Associated With a Higher Proportion of Injuries

**DOI:** 10.3389/fspor.2019.00020

**Published:** 2019-09-06

**Authors:** Pascal Edouard, Hervé Sanchez, Cyprien Bourrilhon, Sébastien Homo, Julien Frère, Johan Cassirame

**Affiliations:** ^1^Inter-University Laboratory of Human Movement Science (LIBM EA 7424), University of Lyon, University Jean Monnet, Saint-Étienne, France; ^2^Sports Medicine Unit, Department of Clinical and Exercise Physiology, Faculty of Medicine, University Hospital of Saint-Étienne, Saint-Étienne, France; ^3^Medical Commission, French Athletics Federation (FFA), Paris, France; ^4^Division de Médecine Physique et Réadaptation, Swiss Olympic Medical Center, Centre de Médecine du Sport, Centre Hospitalier Universitaire Vaudois, Lausanne, Switzerland; ^5^European Athletics Medical & Anti Doping Commission, European Athletics Association, Lausanne, Switzerland; ^6^FIR Division, French Athletics Federation (FFA), Paris, France; ^7^DevAH, Université de Lorraine, Nancy, France; ^8^Faculté des Sciences du Sport, Université de Lorraine, Nancy, France; ^9^EA 4660, Culture, Sport, Health and Society Department and Exercise Performance, Health, Innovation platform, University of Bourgogne France Comté, Besançon, France; ^10^EA 7507, Laboratoire Performance, Santé, Métrologie, Société, Reims, France

**Keywords:** sports injury prevention, biomechanics, pole vault, epidemiology, track and field, top-level athletes, injury risk

## Abstract

**Background:** Pole vault is a highly demanding sport where many physical and technical requirements are engaged in performance process. Considering level of energy transferred from athlete's horizontal speed to the pole during pole bending, we can imagine that associated musculoskeletal tensions, in addition to trials accumulation, can increase the risk of (specific) injuries. Given the multiple morphological, physical and technical characteristics of vaulters and ways of pole vaulting, we can hypothesis that some patterns of pole vaults can lead to higher injury risk than others.

**Aim:** To analyze the potential association between the biomechanical patterns of pole vault and the history of injuries.

**Method:** We conducted a study over national-level pole vaulters including the prospective collection of pole vault biomechanical data during competition at the national elite indoor championship and youth national indoor championship (U17 and U20), associated with the retrospective collection of their injuries during the 12 preceding months through an online questionnaire.

**Results:** Among the 88 pole vaulters participating in these championships, 62 (70.5%) accepted to participated in this study, and their pole vault biomechanical and injury data were collected. 77.4% reported having presented at least one injury during the 12 preceding months. One biomechanical parameter related to the take-off phase (lower H2, i.e., height of the grip (superior) hand from the ground when the athlete subsequently took off from the ground) and some biomechanical parameters related to the terminal phase of the run-up phase (higher Spd [i.e., speed between 10 and 5 meters to the box), SL_adj_ (last stride adjustment), SL_var_ (stride length variation), t_c_ (contact time)] were significantly associated with higher proportions of all injuries.

**Conclusion:** Biomechanical pole vault patterns during the competition day were associated with a higher proportion of history of all injuries. Although the injury data collection was retrospective leading to recall bias risk, and do not allow determining cause-consequence relationships regarding biomechanical patterns and injury occurrence, this present study is the first to analyze potential association between the biomechanical pole vault patterns and injury occurrence, which is of great help to provide hypotheses/ideas to design future studies and to move forward into prevention measures.

## Introduction

Pole vault is a highly demanding specialty of Athletics, in the discipline of jumps (https://www.iaaf.org/disciplines), where many physical and technical requirements are engaged in performance process (Zagorac et al., 2008; Cassirame et al., 2017). This large combination of capabilities needed to perform at best includes for instance running speed, strength and agility, as well as important technical skills (Ekevad and Lundberg, 1997; Frère et al., 2010; Linthorne and Weetman, 2012; Schade and Arampatzis, 2012; Cassirame et al., 2017).

**Figure 1:**
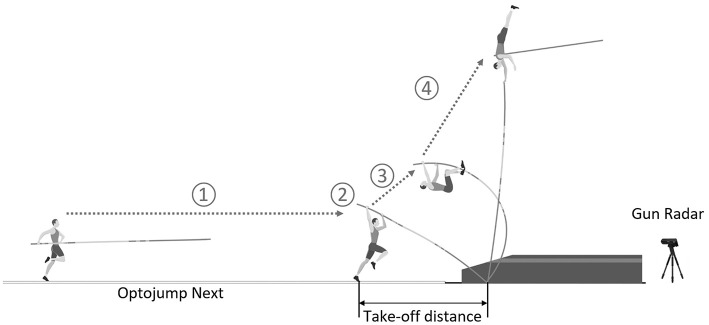
Description of the pole vault trial by the 4 successive phases: (1) run-up, (2) pole planting and take-off, (3) pole bending, and (4) pole straightening and bar clearance (Frère et al., 2010), and of the experimental setup of pole vault biomechanical measurements.

Pole vault training consequently includes physical and technical training, and can be processed differently by each coach considering his own approach of the problem and the individual characteristics of pole vaulters (Gross et al., 2019). Specific technical points can be train in isolation. However, pole vault training often includes a high number of vault trials to improve and optimize the integration of the global pole vault skill/pattern by the vaulter. Pole vault trial is generally described by 4 successive phases: (1) run-up, (2) pole planting and take-off, (3) pole bending, and (4) pole straightening and bar clearance (Figure 1) (Frère et al., 2010). During those phases, the athlete could benefit from the elastic properties of the vaulting pole to gain in mechanical energy and achieve a high performance (Schade et al., 2004). Considering the level of energy transferred from horizontal speed of vaulter to the pole during pole planting, take-off, and pole bending phases (Ekevad and Lundberg, 1997; Frère et al., 2010; Linthorne and Weetman, 2012; Schade and Arampatzis, 2012), we can imagine that such musculoskeletal tensions/constraints can be associated with an increased risk of (specific) injuries, which increase with the accumulation of trials. In addition, given the multiple possibilities of morphological, physical and technical characteristics of the vaulters, and thus, the multiple ways to perform the pole vault, we can hypothesis that some patterns of pole vaults lead to higher injury risk than others.

The pole vault practice indeed bears the risk of injuries (Rebella et al., 2008; Rebella, 2015). An injury rate of 26.4 injuries per 100 athletes per season (95% confidence intervals, 18.6–36.4) and 7.1 injuries per 1,000 athletic-exposures (95% confidence intervals, 5.0–9.8) has been reported in 140 high school pole vaulters aged 16.1 ± 1.2 years (Rebella et al., 2008). In 135 collegiate pole vaulters aged 20.6 ± 1.4 years, Rebella (2015) reported a quite similar injury incidence of 7.9 injuries per 1,000 athlete-exposures, with 15% of injuries leading to season-ending, although the majority of injuries lead to an average time-loss of 9 days (Rebella, 2015). But it is important to note that catastrophic injuries have also been described (Boden et al., 2001, 2012). In other epidemiological studies on injuries in athletics, pole vaulters were included in the groups of jumpers making impossible the distinction of the specific injury risk and characteristics of pole vaulters (Watson and Dimartino, 1987; D'Souza, 1994; Bennell and Crossley, 1996; Edouard et al., 2011, 2015a,b; Jacobsson et al., 2012, 2013).

Although few data are available on pole vaulters' injuries (Rebella et al., 2008; Rebella, 2015), making the need of further studies, the prevention of injuries in pole vaulters seems to be an important challenge for athletes and all stakeholders around them, both in a sports performance and health protection strategies. Better understanding the injury mechanisms and risk factors represents a relevant research direction to move forward into prevention (van Mechelen et al., 1992; Bahr and Krosshaug, 2005). Focusing on injury mechanisms, Rebella (2015) reported that the technique play an important role in the occurrence of injuries: vaulting mechanisms accounted for 67.1% of all injuries, with 32.8% occurring during the plant/take-off phase. Almost all back injuries and majority of shoulder and hamstring injuries occurred during the plant/take-off. These results support that better understanding the biomechanics of pole vault is of interest in this injury prevention perspective.

To date, no study investigated the association between the occurrence of injuries related to pole vault practice and the athletes' characteristics and technical way to vault. Literature (Angulo-Kinzler et al., 1994; Schade et al., 2004) and our own observations during last 10 years of athlete's follow-up highlighted large variabilities in inter-individual characteristics and pole vault mechanical parameters (take-off speed, grip height, pole stiffness, stride regulation, take-off position, …). Given the relationships between pole vault biomechanics and injury mechanisms (Rebella, 2015), it seems of interest to determine whether some pole vault technical and performance determinants would be associated to injuries. In this context, the aim of the present study was to analyze the potential association between the biomechanical patterns of pole vault and the history of injuries. We hypothesized that pole planting and take-off phase parameters can be associated with risk of injuries considering impact and force applied in this moment to initiate energy conversion.

## Methods

### Study Design and Procedure

We conducted a study over national-level pole vaulters including the prospective collection of pole vault biomechanical data during a competition in the context of the national Elite indoor championship and youth national indoor championship (U17 and U20) and the retrospective collection of injuries during the 12 preceding months through an online questionnaire. Those data were collected as part of national Elite follow-up programme from French Athletics Federation (https://www.athle.fr). The study protocol was reviewed and approved by the Saint-Etienne University Hospital Ethics Committee (Institutional Review Board: IORG0007394; IRBN322016/CHUSTE).

### Population

We proposed to all pole vaulters participating at national Elite indoor championship and youth national indoor championship (U17 and U20) to be volunteer for this study. Pole vaulters were included if they were registered with the French Athletics Federation, had no contra-indication for athletics participation, were able to participate at the pole vault competition, were able to read and reply to survey in French sent by internet, and accepted to participate at the study.

The day of the competition, athletes (and their parents when minors) were informed about the study aim and procedure, and gave their consent to participate and their data being used for research.

### Injury Data Collection

At the time of the competition the included pole vaulters were asked to complete an online survey about number of year of pole vault practice, mean number of hours of athletics training per week, if they had an injury history during the last 12 months, and if they have currently a pain or discomfort during pole vault. For the purpose of the study injury was defined as: “Any pain, discomfort, or lesion of the musculoskeletal system (e.g., bones, muscles, tendons, ligaments…), which occurred during sports practice (i.e., training or competition), regardless of the consequences on sport and medical attention, occurring in the last 12 months.” If athletes replied yes, they were asked to detail for each injury the injury location (e.g., hamstring, ankle…).

### Pole Vault Biomechanical Data Acquisition

Pole vault biomechanical data were collected, in the context of a national level competition, during run-up until take-off with the similar set-up than during previous studies (Cassirame et al., 2018). Twenty meters of optoelectronic system (Optojump Next Microgate, Bolzano, Italy) was installed on the official lane to measure run-up kinematics. Due to the landing mat, optoelectronic system could not be installed until the planting box and was installed up until 2.00 or 2.20 m before the box (Figure 1). This material permits measurement of contact time on the floor (t_c_), aerial time (t_a_), stride rate (SR), and stride length (SL). SL asymmetry (SL_asy_) was calculated as the absolute difference of distance covered on three left-foot strides minus the distance covered on three right-foot strides. SL variability (SL_var_) was calculated as the mean of the differences between stride length over successive steps. SR, SL, SL_asy_, SL_var_, t_a_, and t_c_ were measured and averaged from the 3rd up to 8th last stride of the approach. Last two strides of the run-up were not take into account because they are commonly used to adjust take-off distance and are not representative of the running kinematic (Makaruk et al., 2016). Finally, last stride adjustment (SL_adj_) was calculated as the final SL minus the penultimate SL. Negative SL_adj_ indicated a reduction in the last SL, and a positive value indicated a longer final stride (Cassirame et al., 2018). Position of the foot at take-off (PoTk) was calculated using position data output from the optoelectronic system and the distance from the planting box (Figure 1).

Horizontal running velocity was measured using Radar gun (Stalker Pro II, Stalker ltd, Plano, TX) positioned behind the landing mat at 1.4 meter height offering no angle deviation with athletes trajectory (Figure 1). Data output from radar were collected at 46.9 Hz by MookyStalker software (Matsport, Saint-Ismier, France) and synchronized with the Optojump Next system to calculate approach speed between 10 and 5 meters to the box (Spd) and speed increase in last 5 m of the run-up (ΔSpd).

**Figure 2:**
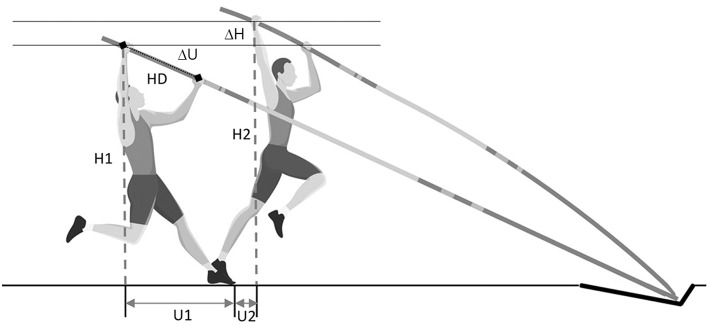
Description of the analysis of the take-off position: Position 1 occurred when the athlete was in contact with the ground at the instant of pole plant in the box. Position 2 occurred at the instance that the athlete subsequently took off from the ground. At both positions, the height of the grip (superior) hand from the ground was measured and noted as H1 and H2 for Positions 1 and 2, respectively. In addition, anteroposterior distance between the grip hand and the take-off foot's toes was calculated at the two positions and noted as U1 and U2. From these four measurements, ΔH and ΔU were calculated in order to obtain vertical and horizontal displacements of the grip hand between the two positions. ΔH and ΔU were calculated as follows: ΔH = H2–H1 and ΔU = U2–U1. The distance between hands (HD) and the distance between the grip hand and pole extremity was also measured.

During the take-off phase, a video analysis was performed with a Gopro Hero 5 camera (San Mateo, California, United-States) using a sampling rate of 240 frames per seconds and a resolution of 1280 ×720 pixels. The camera was positioned at a distance of 4 m perpendicular to the lane at a 3.5 m distance from the box to avoid parallax error. Before each competition, calibration videos were collected using a calibration stick of known length (2.40 m) in the plane of measurement. Video analyzes were manually performed with Kinovea software 08.15 (Joan Charmant & Contributors, Bordeaux, France) to output several length measurements in two different positions. The Position 1 occurred when the athlete was in contact with the ground at the instant of pole plant in the box, and the Position 2 occurred when the athlete subsequently took off from the ground (Figure 2). At both positions, the height of the grip (superior) hand from the ground was measured and noted as H1 and H2 for Positions 1 and 2, respectively. In addition, the anteroposterior distance between the grip hand and the take-off foot's toes was calculated at the two positions and noted as U1 and U2 (Figure 2). If the grip hand was posterior to the toes, this value was negative. From these four measurements, ΔH and ΔU were calculated in order to obtain vertical and horizontal displacements of the grip hand between the two positions. ΔH and ΔU were calculated as follows: ΔH = H2–H1 and ΔU = U2–U1. To complete this analysis, the distance between hands (HD) and the distance between the grip hand and pole extremity was also measured. This last measurement permits to calculate the grip height (Grip) used by athlete during trials.

Finally, data related to the poles used during the competition were collected from coach and/or athlete interview. For each trial, pole length and stiffness index (P_Stiff_) were collected. Pole length information was use to deduct grip (Grip) used by athlete use measurement processed by video analysis (Distance upper hand to extremity of the pole in the box).

### Data Analyzes

Descriptive analyzes were performed with the total population, and separated into female and male pole vaulters, and then divided according to age categories (youth, junior and adult), using frequency with percentages [and 95% Confidence Intervals (95%CI)] for categorical data, and mean and standard deviations (± SD) for continuous variables. Normal distribution of the data was checked by the Shapiro-Wilk normality test. A two-way (sex × age category) ANOVA was performed to analyze the potential differences in pole vault biomechanical parameters according to these factors. A Chi^2^ test was used to compare injured pole vaulters' proportions according to sex and age category.

In order to analyze the association between pole vault biomechanics and history of injuries (outcomes were: all injuries, and the main reported injury location: hamstring injuries, quadriceps injuries, ankle injuries, upper extremity injuries, and pain when practicing pole vault), we used a logistic stepwise regression model including several explanatory variables selected after collinearity analysis (Spd, ΔSpd, SL, SR, t_a_, t_c_, SL_adj_, SL_asy_, SL_var_, P_Stiff_, Grip, PoTk, HD, H1, U1, H2, U2, ΔH, ΔU) and adjusted for sex and age category. The significance level was set at *P* <0.05. Analyzes were performed using Excel (Office, Microsoft®, 2017) and JASP (JASP Team software, Version 0.8.5.1, University of Amsterdam, Netherlands).

## Results

### Population

**Figure 3:**
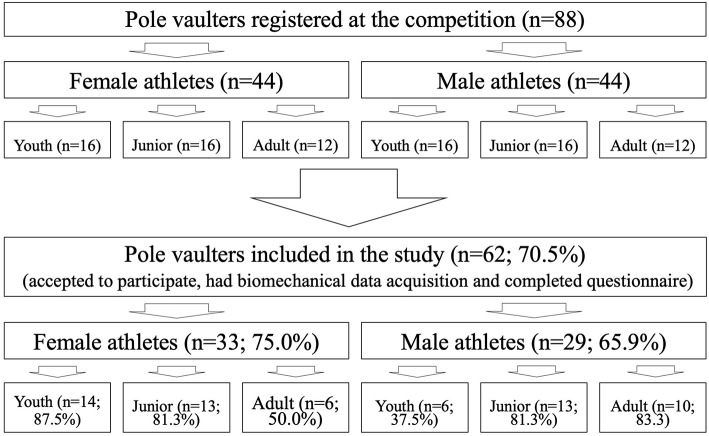
Flow chart of the population inclusion.

Among the 88 pole vaulters registered at the competition, 62 (70.5%) accepted to participate in the present study, had pole vault biomechanical data acquisition, completed the online questionnaire, and were thus included in the present study. The flow chart of the included population is presented in Figure 3, and the characteristics of the population in Table 1.

**Table 1:** Characteristics of the included pole vaulters with regards to pole vault practice and biomechanics and history of injuries.

	**Total**	**Female athletes**			**Male athletes**			**Sex**	**Age category**	**Sex x age category**
		**Youth**	**Junior**	**Adult**	**Youth**	**Junior**	**Adult**			
	***n* = 62**	***n* = 14**	***n* = 13**	***n* = 6**	***n* = 6**	***n* = 13**	***n* = 10**			
**History of pole vault practic**											
Number of years of practice (years)	6.69 ± 4.47	3.9 ± 1.8	4.4 ± 1.2	10.8 ± 4.2	4.8 ± 1.3	6.2 ± 1.7	12.9 ± 6.1	*F*_(1, 56)_ = 3.435; *p* = 0.069	***F***_**(2, 56)**_ **=** **28.855;** ***p<*** **0.001**	*F*_(2, 56)_ = 0.182; *p* = 0.834
Training per week (hours)	9.53 ± 4.73	7.6 ± 3.2	8.0 ± 2.9	13.0 ± 1.8	6.1 ± 2.5	9.8 ± 4.4	13.9 ± 7.0	*F*_(1, 56)_ = 0.117; *p* = 0.734	***F***_**(2, 56)**_ **=** **10.723;** ***p<*** **0.001**	*F*_(2, 56)_ = 0.796; *p* = 0.456
**Pole vault biomechanics**											
**Run-up phase**										
Spd (m/s)	7.81 ± 0.86	6.8 ± 0.5	7.3 ± 0.3	7.7 ± 0.1	8.2 ± 0.1	8.5 ± 0.3	9.0 ± 0.2	***F***_**(1, 56)**_ **=** **239.019;** ***p<*** **0.001**	***F***_**(2, 56)**_ **=** **25.743;** ***p<*** **0.001**	*F*_(2, 56)_ = 0.596; *p* = 0.555
ΔSpd (m/s)	0.13 ± 0.14	0.10 ± 0.11	0.11 ± 0.10	−0.6 ± 0.04	0.21 ± 0.14	0.28 ± 0.11	0.11 ± 0.10	***F***_**(1, 56)**_ **=** **29.401;** ***p<*** **0.001**	***F***_**(2, 56)**_ **=** **12.904;** ***p<*** **0.001**	*F*_(2, 56)_ = 0.571; *p* = 0.568
SL (cm)	192.95 ± 16.62	176.5 ± 12.2	182.5 ± 9.9	197.5 ± 12.2	190.2 ± 8.6	203.2 ± 7.7	215.2 ± 7.0	***F***_**(1, 56)**_ **=** **44.985;** ***p<*** **0.001**	***F***_**(2, 56)**_ **=** **23.168;** ***p<*** **0.001**	*F*_(2, 56)_ = 0.673; *p* = 0.514
SR (stride/s)	3.94 ± 0.22	3.8 ± 0.1	3.8 ± 0.2	3.8 ± 0.2	4.1 ± 0.1	4.1 ± 0.2	4.2 ± 0.2	***F***_**(1, 56)**_ **=** **59.615;** ***p<*** **0.001**	*F*_(2, 56)_ = 1.967; *p* = 0.149	*F*_(2, 56)_ = 0.020; *p* = 0.980
t_a_ (s)	0.12 ± 0.01	0.12 ± 0.01	0.12 ± 0.01	0.13 ± 0.01	0.12 ± 0.01	0.12 ± 0.01	0.12 ± 0.01	*F*_(1, 56)_ = 1.601; *p* = 0.211	*F*_(2, 56)_ = 2.146; *p* = 0.126	*F*_(2, 56)_ = 1.319; *p* = 0.276
t_c_ (s)	0.13 ± 0.01	0.14 ± 0.01	0.14 ± 0.01	0.13 ± 0.00	0.12 ± 0.1	0.12 ± 0.1	0.12 ± 0.1	***F***_**(1, 56)**_ **=** **22.878;** ***p<*** **0.001**	***F***_**(2, 56)**_ **=** **4.145;** ***p=*** **0.021**	*F*_(2, 56)_ = 1.284; *p* = 0.285
SL_adj_ (cm)	−13.87 ± 13.10	−16.9 ± 12.6	−6.3 ± 14.0	−17.8 ± 11.5	−14.6 ± 7.3	−11.1 ± 12.4	−20.3 ± 14.1	*F*_(1, 56)_ = 0.229; *p* = 0.634	***F***_**(2, 56)**_ **=** **3.587;** ***p=*** **0.034**	*F*_(2, 56)_ = 0.401; *p* = 0.671
SL_asy_ (cm)	−0.41 ± 8.58	−2.2 ± 10.1	0.8 ± 8.6	2.6 ± 8.6	1.2 ± 12.2	−0.4 ± 5.6	−2.2 ± 8.3	*F*_(1, 56)_ = 0.122; *p* = 0.728	*F*_(2, 56)_ = 0.039; *p* = 0.962	*F*_(2, 56)_ = 0.888; *p* = 0.417
SL_var_ (cm)	8.33 ± 4.51	9.8 ± 5.2	7.4 ± 4.4	9.4 ± 6.2	9.3 ± 4.4	6.9 ± 3.7	8.1 ± 3.8	*F*_(1, 56)_ = 0.410; *p* = 0.525	*F*_(2, 56)_ = 1.545; *p* = 0.222	*F*_(2, 56)_ = 0.031; *p* = 0.969
**Pole planting and take-off phase**										
P_Stiff_ (cm/50 lb)	21.10 ± 3.71	22.9 ± 2.1	23.3 ± 3.6	24.8 ± 1.7	21.2 ± 3.4	18.8 ± 2.1	16.4 ± 1.4	***F***_**(1, 56)**_ **=** **51.947;** ***p<*** **0.001**	*F*_(2, 56)_ = 1.370; *p* = 0.262	***F***_**(2, 56)**_ **=** **6.994;** ***p=*** **0.002**
Grip (m)	4.24 ± 0.38	3.8 ± 0.2	4.0 ± 0.2	4.1 ± 0.1	4.4 ± 0.3	4.6 ± 0.1	4.8 ± 0.1	***F***_**(1, 56)**_ **=** **216.716;** ***p<*** **0.001**	***F***_**(2, 56)**_ **=** **16.142;** ***p<*** **0.001**	***F***_**(2, 56)**_ **=** **0.744;** ***p=*** **0.048**
PoTk (m)	3.26 ± 0.46	2.8 ± 0.3	2.9 ± 0.2	3.1 ± 0.2	3.5 ± 0.2	3.7 ± 0.2	3.8 ± 0.2	***F***_**(1, 56)**_ **=** **142.881;** ***p<*** **0.001**	***F***_**(2, 56)**_ **=** **6.210;** ***p=*** **0.004**	*F*_(2, 56)_ = 0.511; *p* = 0.603
HD (cm)	60.95 ± 9.51	61.1 ± 8.3	55.2 ± 7.7	64.3 ± 5.1	59.7 5.6	58.4 ± 12.8	70.3 ± 4.7	*F*_(1, 56)_ = 1.300; *p* = 0.259	***F***_**(2, 56)**_ **=** **7.180;** ***p=*** **0.002**	*F*_(2, 56)_ = 0.766; *p* = 0.470
H1 (cm)	187.98 ± 22.03	183.9 ± 8.0	172.4 ± 23.4	187.7 ± 8.6	201.7 1.8	183.8 ± 29.8	211.3 ± 10.5	***F***_**(1, 56)**_ **=** **12.149;** ***p<*** **0.001**	***F***_**(2, 56)**_ **=** **7.097;** ***p=*** **0.002**	*F*_(2, 56)_ = 0.525; *p* = 0.595
U1 (cm)	−39.68 ± 18.01	−42.6 ± 17.0	−40.9 ± 11.5	−46.1 ± 16.6	−44.7 ± 26.4	−26.5 ± 15.2	−44.3 ± 20.5	*F*_(1, 56)_ = 1.004; *p* = 0.321	*F*_(2, 56)_ = 2.763; *p* = 0.072	*F*_(2, 56)_ = 1.342; *p* = 0.270
H2 (cm)	196.34 ± 21.17	193.0 ± 7.1	184.8 ± 20.8	194.9 ± 12.3	209.5 ± 6.0	190.0 ± 31.3	217.1 ± 10.6	***F***_**(1, 56)**_ **=** **8.351;** ***p=*** **0.005**	***F***_**(2, 56)**_ **=** **5.548;** ***p=*** **0.006**	*F*_(2, 56)_ = 1.096; *p* = 0.341
U2 (cm)	−22.79 ± 12.88	−25.5 ± 10.4	−29.5 ± 13.4	−24.1 ± 9.7	−21.1 ± 20.4	−13.5 ± 11.0	−22.7 ± 9.0	***F***_**(1, 56)**_ **=** **4.848;** ***p=*** **0.032**	*F*_(2, 56)_ = 0.165; *p* = 0.848	*F*_(2, 56)_ = 2.100; *p* = 0.132
ΔH (cm)	16.89 ± 10.86	17.1 ± 10.6	11.4 ± 7.5	22.0 ± 8.2	23.6 ± 11.8	13.0 ± 9.9	21.7 ± 13.3	*F*_(1, 56)_ = 0.841; *p* = 0.363	***F***_**(2, 56)**_ **=** **5.339;** ***p=*** **0.008**	*F*_(2, 56)_ = 0.484; *p* = 0.619
ΔU (cm)	8.36 ± 7.95	9.1 ± 4.1	12.4 ± 15.3	7.2 ± 4.6	7.8 ± 5.6	6.2 ± 2.7	5.9 ± 3.4	*F*_(1, 56)_ = 1.902; *p* = 0.173	*F*_(2, 56)_ = 0.606; *p* = 0.549	*F*_(2, 56)_ = 0.673; *p* = 0.514
**History of injuries**											
**Proportion of injured pole vaulters during the 12 preceding months (95%CI)**										
All injuries	77.4 (±10.4)	64.3 (±25.1)	84.6 (±19.6)	100.0 (±0.0)	66.7 (±37.7)	84.6 (±19.6)	70.0 (±28.4)	Chi^2^ = 0.076; *p* = 0.783	Chi^2^ = 2.269; *p* = 0.263	
Hamstring	22.6 (±10.4)	14.3 (±18.3)	23.1 (±22.9)	66.7 (±37.7)	30.8 (±25.1)	10.0 (±18.6)	17.2 (±13.7)	Chi^2^ = 0.888; *p* = 0.346	Chi^2^ = 2.779; *p* = 0.249	
Quadriceps	9.7 (±7.4)	21.4 (±21.5)	7.7 (±14.5)	16.7 (±29.8)	0.0 (±0.0)	7.7 (±14.5)	3.4 (± 6.6)	Chi^2^ = 2.419; *p* = 0.120	Chi^2^ = 0.980; *p* = 0.612	
Ankle	17.7 (±9.5)	14.3 (±18.3)	23.1 (±22.9)	0.0 (±0.0)	16.7 (±29.8)	7.7 (±14.5)	40.0 (±30.4)	Chi^2^ = 0.324; *p* = 0.569	Chi^2^ = 0.780; *p* = 0.677	
Upper extremity	6.5 (±6.1)	7.1 (±13.5)	0.0 (±0.0)	0.0 (±0.0)	16.7 (±29.8)	7.7 (±14.5)	10.0 (±11.1)	Chi^2^ = 1.368; *p* = 0.242	Chi^2^ = 0.711; *p* = 0.701	
Proportion of pole vaulters with pain during practice (%)	4.8 (±5.3)	0.0 (±0.0)	0.0 (±0.0)	0.0 (±0.0)	0.0 (±0.0)	0.0 (±0.0)	30.0 (±28.4)	Chi^2^ = 3.587; *p* = 0.058	**Chi**^**2**^ **=** **9.064;** ***p=*** **0.011**	

*Spd, speed between 10 and 5 meters to the box; ΔSpd, speed increase in last 5 meter of the run-up; SL, stride length; SR, stride rate; t_a_, aerial time; t_c_, contact time; SL_adj_, last stride adjustment; SL_asy_, stride length asymmetry; SL_var_, stride length variation; P_Stiff_, pole length and stiffness; Grip, distance upper hand to extremity of the pole in the box; PoTk, position of the foot at take-off; HD, distance between hands; H1, height of the superior hand from the ground at Position 1; U1, antero-posterior displacement of the superior hand from the take-off foot's toes at Position 1; H2, height of the grip (superior) hand from the ground at Position 2; U2, antero-posterior displacement of the superior hand from the take-off foot's toes at Position 2; ΔH, vertical distance traveled by the superior hand between the two positions; ΔU, horizontal distance traveled by the superior hand between the two positions; 95%CI, 95% confidence intervals. Significant differences are highlighted in bold*.

### History of Injuries

On the 62 pole vaulters, 48 (77.4%) reported having presented at least one injury during the 12 preceding months. Within these 48 athletes, 29 (60.4%) presented with one injury, 14 (29.2%) presented with two, 2 (4.2%) with three, 2 (4.2%) with four, and one (2.1%) with five injuries. Proportions of injured pole vaulters according to injury location are reported in Table 1. 4.8% reported having pain during pole vault practice, they are all male adult athletes (Table 1).

### Pole Vault Biomechanical Parameters

Pole vault biomechanics differed between sex for many parameters: Spd, ΔSpd, SL, SR, t_c_, P_Stiff_, Grip, PoTk, H1, H2, U2; as well as between age category: Spd, ΔSpd, SL, t_c_, SL_adj_, Grip, PoTk, HD, H1, H2, ΔH; with sex × age interaction for P_Stiff_ and Grip (Table 1).

### Pole Vault Biomechanical Parameters and History of Injuries

Results of the logistic regressions are presented in Table 2. H2, training time per week, SL_adj_, Spd, t_c_, and SL_var_ were significantly associated with history of all injuries [although the model was not significant (*p* = 0.067)]. Duration of training per week and ΔSpd were significantly associated with history of ankle injuries [although the model was not significant (*p* = 0.141)]. Logistic regressions were not significant for history of hamstring injuries, and have been not performed for quadriceps injuries, upper extremity injuries and pain when practicing pole vault, due to the small number of injuries in these respective categories.

**Table 2:** Results of the logistic regressions (stepwise multiple regression model) analyzing the association between pole vault biomechanics and history of injuries (outcomes were: all injuries, hamstring injuries, ankle injuries).

**Models Summaries**									
**Model**	**Number of the model in the stepwise regression**	**Deviance**	**AIC**	**BIC**	***p***	**Nagelkerke R^2^**	**AUC**	**Sensitivity**	**Specificity**
History of all injuries	9	35.98	53.978	73.123	0.067	0.588	0.926	0.938	0.643
History of hamstring injuries	3	60.65	66.653	73.034	0.143	0.131	0.693	0.071	0.979
History of ankle injuries	5	43.38	53.379	64.014	0.141	0.345	0.854	0.273	0.941
**Coefficients**									
**Model**	**Number of the model in the stepwise regression**	**Parameter**	**Estimate**	**Standard Error**	**Odds Ratio**	***z***	***p***	**95% CI lower bound**	**95% CI upper bound**
History of all injuries	9	(Intercept)	−30.636	17.798	4.955e−14	−1.721	0.085	−65.519	4.248
		**H2**	**−0.202**	**0.069**	**0.817**	**−2.931**	**0.003**	**−0.338**	**−0.067**
		**Training per week**	**0.397**	**0.156**	**1.488**	**2.540**	**0.011**	**0.091**	**0.704**
		**SL**_**adj**_	**0.158**	**0.067**	**1.172**	**2.349**	**0.019**	**0.026**	**0.290**
		**ΔSpd**	**4.278**	**1.550**	**72.110**	**2.759**	**0.006**	**1.239**	**7.317**
		**t**_**c**_	**276.336**	**114.909**	**1.026e+120**	**2.405**	**0.016**	**51.119**	**501.553**
		**SL**_**var**_	**0.321**	**0.139**	**1.378**	**2.304**	**0.021**	**0.048**	**0.594**
		ΔH	0.104	0.053	1.110	1.953	0.051	−0.000	0.208
		Acc	−7.977	4.748	3.432e−4	−1.680	0.093	−17.284	1.329
History of hamstring injuries	3	**(Intercept)**	**−9.475**	**4.720**	**7.677e−5**	**−2.007**	**0.045**	**−18.726**	**−0.223**
		t_a_	61.247	37.447	3.975e +26	1.636	0.102	−12.147	134.641
		Number of years of practice	0.094	0.063	1.099	1.491	0.136	−0.030	0.218
History of ankle injuries	5	**(Intercept)**	**−9.473**	**2.667**	**7.687e** **−5**	**−3.552**	**<.001**	**−14.700**	**−4.247**
		**Training per week**	**0.228**	**0.091**	**1.255**	**2.500**	**0.012**	**0.049**	**0.406**
		**ΔSpd**	**8.709**	**3.657**	**6058.275**	**2.381**	**0.017**	**1.541**	**15.877**
		U2	−0.063	0.032	0.939	−1.954	0.051	−0.126	0.000
		ΔH	0.066	0.043	1.068	1.550	0.121	−0.017	0.150

*Presence of the injury problem coded as class 1. Spd, speed between 10 and 5 meters to the box; ΔSpd, speed increase in last 5 meter of the run-up; SL, stride length; SR, stride rate; t_a_, aerial time; t_c_, contact time; SL_adj_, last stride adjustment; SL_asy_, stride length asymmetry; SL_var_, stride length variation; P_Stiff_, pole length and stiffness; Grip, distance upper hand to extremity of the pole in the box; PoTk, position of the foot at take-off; HD, distance between hands; H1, height of the superior hand from the ground at Position 1; U1, antero-posterior displacement of the superior hand from the take-off foot's toes at Position 1; H2, height of the superior hand from the ground at Position 2; U2, antero-posterior displacement of the superior hand from the take-off foot's toes at Position 2; ΔH, vertical distance traveled by the superior hand between the two positions; ΔU, horizontal distance traveled by the superior hand between the two positions; 95% CI, 95% confidence intervals; AIC, Aikaike criterion; BIC, Bayes criterion; AUC, Area Under the Curve. Significant differences are highlighted in bold*.

## Discussion

The main findings of the present study were that some biomechanical pole vault parameters were associated with a higher proportion of history of all injuries. Parameters related to the take-off phase (lower H2) and to the terminal phase of the run-up phase (higher Spd, SL_adj_, SL_var_, t_c_), as well as higher volume of training per week, were associated with a higher proportion of history of all injuries. These findings partially confirm our hypothesis. We hypothesized that pole planting and take-off phase parameters can be associated with risk of injuries considering impact and force applied in this moment to initiate energy conversion. Our results reported that one biomechanical parameter related to the take-off phase and some biomechanical parameters related to the terminal phase of the run-up phase (and preparation of the planting/take-off phase) were significantly associated with a higher proportion of history of all injuries. However, given the retrospective design of the injury data collection, it is not possible to conclude about the cause or consequence of the present biomechanical parameters with regards to their role in the injury occurrence.

### Horizontally-Based Vaulting Techniques Associated With Injuries

Our present results reported that a lower H2 [i.e., height of the grip (superior) hand from the ground when the athlete took off from the ground], a higher stride length adjustment (i.e., a less shorter last stride relative to the penultimate one), and a higher horizontal speed (between 10 and 5 m from the planting box), were significantly associated with a higher proportion of history of all injuries. All together, these three predictors reflect a horizontally-based vaulting technique. Indeed, the lesser adjustment in stride length among the two last strides tends to reduce the possibility of reorienting the athlete's velocity toward a higher vertical component. This might lower the value of H2, and cumulated with a high horizontal velocity, generate a highly horizontally and forward oriented take-off. Such a take-off pattern might lower the pole-ground and take-off angle, which likely increase the injury risk (or this could also be the consequence of previous injuries). Indeed, a more horizontal pole reaction force opposed to the athlete may increase the energy dissipated (as heat) within the hyperextended vaulter's body (Linthorne, 2000), and may elongate tissue above their own elastic capabilities. Gainor et al. (1983) found that such mechanisms could be related to back injuries. Back injuries were not one of the main reported injuries in our present studies (only 4 athletes reported having had back injuries during the 12 preceding months, therefore this injury had not been reported as an outcome). But, our results also suggest that such mechanisms would be related with higher proportions of injuries (either a cause or a consequence). Consequently, we can suggest that producing a higher pole-ground and take-off angle could help to decrease this jerk from the pole, and could be a way to prevent/limit the injury risk (or their secondary compensation). In addition, this suggested strategy to reduce injury risk might not be detrimental for the performance, as Arampatzis et al. (1999) found that the best world-class pole vaulters where those who had the highest values of H2. Although, this has not been proven in our present study. Based on these arguments, we can suggest this action as a win-win performance-prevention strategy. In addition, this reinforce the importance of this transitional phase between running and vaulting, and the importance of a very good mastering of the pole vault technique in order to benefit of the energy from run-up phase, and not to undergo this energy with could be a way to incur injury. Nevertheless, given the retrospective nature of the injury data collection, it is not possible to conclude whether this parameter is a cause or a consequence of the injury. Our discussion is thus only an assumption which should be confirmed in future studies.

### Training Exposure Should Be at the Center of Attention

Higher number of hours per week spent at training was also associated with a higher proportion of history of all injuries. Training volume per week is also related to the level of practice, and a sign of engagement in the pole vault discipline. Pole vaulting practice and training associated induce many mechanical traumatisms by vault itself, but also by typical exercises used to developed athletes' capabilities. Increased volume of training can also improve numbers of traumatisms and stress on the body during those work phases and generated injuries as already reported in other sports (Damsted et al., 2018; Sugimoto et al., 2019). This result seems quite obvious as a higher exposition to the risk logically can lead to higher rate of the problem. This reinforces the need of using values of injuries reported to the exposure (e.g., number of injuries per 1,000 h of practice) (Nielsen et al., 2019). For practical implication, pole vaulters with high training volume should be at the center of attention in order to limit the occurrence of injuries. Since training is fundamental to improve performance, we do not (never) say that it is needed to limit training to prevent injuries. We think that it is needed to find an optimal balance in training volume and intensity (training load), for instance paying attention to pain and/or fatigue, allowing recovery, in order to promote performance (Soligard et al., 2016).

### Perturbation of Running Patterns as a Consequence of Previous Injuries

As previously discussed, future studies should confirm the latter assumptions, since injuries were retrospectively collected and it is not possible to conclude whether these parameters associated with injuries are a cause or a consequence of the injury. However, some associated parameters could be hypothesized as consequences of the injuries. Our results reported that higher contact time (t_c_) was associated with a higher proportion of history of all injury. This is in agreement with results from Mann et al. (2015) reporting increased in running contact time in runners with previous injuries compared to healthy control runners. In addition, we observed that stride length variability (SL_var_) was associated with the injuries history outcome. An increased stride variability could also be a consequence of lateralized injuries and higher neuro-muscular control to compensate disorder caused by previous injuries (Donoghue et al., 2008).

### Specific Injuries According to Specific Pole Vault Biomechanical Patterns

Regression models of history of hamstring and ankle injuries related with biomechanical patterns are presented in Table 2. For ankle injuries, the model was not significant (*p* = 0.141) and can explain 35% of the variance, although training per week and speed increased in the last 5 meters were significantly associated with higher proportion of ankle injury history. As discussed previously for all injuries, it seems that higher engagement in pole vault would be associated with higher ankle injury history. For history of hamstring injuries, although there were no significant association, larger inertial loads during high speed running tended to be related to history of hamstring injuries (Chumanov et al., 2011). Higher aerial time reported in our present study could be related with this aspect. Increasing the aerial time would mind increasing the swing phase, and thus potentially the end of the swing phase, which has been reported as associated to hamstring injuries (Chumanov et al., 2011; Kenneally-Dabrowski et al., 2019). The models were not significant, and did not report significant association between biomechanical parameters and history of injuries for hamstring injuries. Moreover, the number of observations were small. Thus, it is therefore impossible to conclude of the association. Nevertheless, we would like to discuss some assumptions regarding these preliminary results in order to provide some perspectives for future researches since the present insignificant results are in agreement with some previous findings. Indeed, Although we reported some differences in pole vault biomechanical parameters between sex, in agreement with previous study (Schade et al., 2004; Cassirame et al., 2017), and age categories, it seems that these latter parameters did not influence the proportion of history of injuries, as shown in the parameters revealed as significant in the regression models.

### Methodological Considerations

As strength, this study is the first analyzing biomechanical data together with injury data in pole vault, with the goal of better understanding injury risk factors and mechanics.

Regarding limitation, we can acknowledge the small number of pole vaulters included. However, this was high level pole vaulters (participating in the national championships) and represented 71% of the targeted population. The small sample size lead to a small number of some injury diagnoses (e.g., quadriceps and upper extremity injuries), which did not allow performing regression logistic analyzes. We performed and presented logistic regressions for all secondary outcomes (i.e., hamstring and ankle injuries), although number of observations were small, and logistic regression results showed low *R*^2^ and were not significant. The number of explanatory variables could be considered as too important in comparison to the number of observations. We did not collect anthropomorphic parameters (height and body mass), which would have been of interest to adjust biomechanical parameters. There was a high inter-subject variability in biomechanical parameters, especially in junior. The injury data collection was retrospective leading to the risk of recall bias, and do not allow to determine the cause-consequence relationships regarding the biomechanical pattern and the injury occurrence. Finally, since injury is multifactorial (Bittencourt et al., 2016), other parameters than sex, age category, and pole vault biomechanics should be taken into account to try to reach the optimal approach of injury understanding. All these limitations represent perspectives of future researches, including a prospective data collection of injuries in association with the data collection of biomechanical pole vault parameters, and other parameters which can influence the injury occurrence.

### Practical Implications

Pole vault practice is a sport requiring many physical and technical abilities to create and exchange energy with pole to maximize performance (Ekevad and Lundberg, 1997; Frère et al., 2010; Linthorne and Weetman, 2012; Schade and Arampatzis, 2012; Cassirame et al., 2017). During energetic exchange, especially at take-off, many mechanical constraints are applying on musculoskeletal system. Those constraints are generated by the impact and the long force moment from beginning of pole bending until toes off. During this crucial phase for performance (Linthorne, 2000), many parameters (e.g., running speed) are from one side beneficial for performance and in other side potentially harmful. In addition, body position of athlete and pole vault pattern used by the athlete can produce more or less traumatisms.

Therefore, we think that specific attention should be done for each pole vaulter given its specific pole vault pattern. Performance-prevention management should be a win-win strategy based on individual management. Given the importance of the position of the grip (superior) hand at the tack-off related to the risk of injuries, we can suggest at a practical prevention measure to train athletes to increase the angle between the pole and the horizontal axis at the take-off phase. Mastering the transitional phase between the run-up and the take-off phase should also be at the center of training activities. An optimal balance in training volume and intensity (training load) should be found and pole vaulters with high training exposure should be at the center of attention. Finally, the technical training of pole vaulters with previous injuries should be improved by taking attention to potential compensation.

## Conclusions

Our present results reported that one biomechanical parameter related to the take-off phase (lower H2) and some biomechanical parameter related to the terminal phase of the run-up phase (higher Spd, SL_adj_, SL_var_, t_c_) were significantly associated with higher proportions of all injuries. Although the injury data collection was retrospective leading to the risk of recall bias, and do not allow to determine the cause-consequence relationships regarding the biomechanical patterns and the injury occurrence, this present study is the first to analyze potential association between the biomechanical pole vault patterns and injury occurrence, which is of great help to provide hypotheses/ideas to design future studies and to move forward into prevention measures.

## Data Availability

The datasets generated for this study will not be made publicly available because they are included in a preliminary database.

## Author Contributions

PE and JC conceived, analyzed the data, drafted the manuscript and prepared the table/figure, and designed the study. JC, HS, and SH performed experimentation and data collection. PE, JF, and JC interpreted the results. PE, HS, CB, SH, JF, and JC edited, critically revised the manuscript, and approved the final version.

### Conflict of Interest Statement

The authors declare that the research was conducted in the absence of any commercial or financial relationships that could be construed as a potential conflict of interest.
